# GDCL-NcDA: identifying non-coding RNA-disease associations via contrastive learning between deep graph learning and deep matrix factorization

**DOI:** 10.1186/s12864-023-09501-3

**Published:** 2023-07-27

**Authors:** Ning Ai, Yong Liang, Haoliang Yuan, Dong Ouyang, Shengli Xie, Xiaoying Liu

**Affiliations:** 1 Peng Cheng Laboratory, Shenzhen, 518005 Guangdong China; 2 School of Computer Science and Engineering, Macau University of Science and Technology, Avenida Wai Long, Taipa, China; 3 Pazhou Laboratory (Huangpu), Guangzhou, 510555 Guangdong China; 4 School of Automation, Guangdong University of Technology, Guangzhou, 510006 Guangdong China; 5 Institute of Intelligent Information Processing, Guangdong University of Technology, Guangzhou, 510000 Guangdong China; 6 Computer Engineering Technical College, Guangdong Polytechnic of Science and Technology, Zhuhai, Guangdong 519090 China

**Keywords:** Non-coding RNA-disease associations, Multi-source heterogenous networks, Contrastive learning, Deep graph learning, Deep matrix factorization

## Abstract

Non-coding RNAs (ncRNAs) draw much attention from studies widely in recent years because they play vital roles in life activities. As a good complement to wet experiment methods, computational prediction methods can greatly save experimental costs. However, high false-negative data and insufficient use of multi-source information can affect the performance of computational prediction methods. Furthermore, many computational methods do not have good robustness and generalization on different datasets. In this work, we propose an effective end-to-end computing framework, called GDCL-NcDA, of deep graph learning and deep matrix factorization (DMF) with contrastive learning, which identifies the latent ncRNA-disease association on diverse multi-source heterogeneous networks (MHNs). The diverse MHNs include different similarity networks and proven associations among ncRNAs (miRNAs, circRNAs, and lncRNAs), genes, and diseases. Firstly, GDCL-NcDA employs deep graph convolutional network and multiple attention mechanisms to adaptively integrate multi-source of MHNs and reconstruct the ncRNA-disease association graph. Then, GDCL-NcDA utilizes DMF to predict the latent disease-associated ncRNAs based on the reconstructed graphs to reduce the impact of the false-negatives from the original associations. Finally, GDCL-NcDA uses contrastive learning (CL) to generate a contrastive loss on the reconstructed graphs and the predicted graphs to improve the generalization and robustness of our GDCL-NcDA framework. The experimental results show that GDCL-NcDA outperforms highly related computational methods. Moreover, case studies demonstrate the effectiveness of GDCL-NcDA in identifying the associations among diversiform ncRNAs and diseases.

## Introduction

According to a central dogma of molecular biology, it describes how genetic information is transmitted through RNA to the corresponding protein. Non-coding RNAs (ncRNAs) are a large segment of the transcriptome that do not have apparent protein-coding roles, which are functional RNAs molecule that is not translated into a protein [1]. Thus, for the past decades, there are a view that ncRNAs are transcriptional noise [2]. Until a breakthrough in biotechnology, ncRNAs catch extensive attention of many researchers and completely change the view of biological scientists on RNA function [3]. Hundred studies find that ncRNAs occupy a vital position in life activities by being a key regulators of gene expression, involving the occurrence and development of many diseases and so on [4]. Nowadays, microRNAs (miRNAs), circular RNAs (circRNAs) and long noncoding RNAs (lncRNAs) are commonly studied in disease-associated ncRNAs [3, 5].

More than 1, 500 miRNAs found in the human genome up to now. They have a length of about 21-23 nucleotides, and each miRNA has hundreds of targeted mRNAs. miRNAs are involved in almost every process in human cells. Therefore, researchers believe that every disease is influenced by a miRNAs component [6]. miR-140-5p and miR-146a can target Sirt2, Nrf2, TAF9b/P53, and other pathways, that they take a important place in doxorubicin-induced cardiotoxicity [7, 8]. In the last few years, great efforts be made to discover the latent miRNA-disease associations. For instance, Chen et al. [9] use the matrix decomposition method to discover the disease-associated miRNAs. Peng et al. [10] construct a HN of miRNA-gene-disease with their similarity networks. Auto-encoder (AE) and convolutional neural network (CNN) are used to recognize the characteristic combination and predict the final label for each pair of miRNA and disease, respectively. Jiang et al. [11] design the similarity kernel fusion (SKF) method to integrate diverse similarity kernels of miRNA and disease, which can be more effectively for predicting miRNA-disease associations. Li et al. [12] combine the linear and non-linear features from miRNA and disease to find the latent associations, where the linear features are formed by the correlation profiles of disease-lncRNA and miRNA-lncRNA and the nonlinear features are extracted by graph attention network (GAT).

circRNAs can act as miRNA, or protein inhibitors, which attracts an increasing number of attentions from researchers [13]. They have a closed single-strand continuous circular form. Without 3’ or 5’ polyadenylated tails, they can be resistant to extracellular enzyme-mediated degradation [14]. In Crohn’s disease, hsa$\_$circRNA$\_$103765 can impact tumor necrosis factor-$\alpha$ via cell apoptosis induced [15]. Lei et al. [16] develop a computational path weighted method for inferring circRNA-disease associations by integrating similarity networks and interaction network. More specifically, they calculate a linkage score for each pair of circRNA and disease based on paths linking them. Wei et al. [17] reconstruct the association matrix between circRNAs and diseases based on diverse similarity networks, and use it as a basis for the links prediction task by nonnegative matrix factorization. Wang et al. [18] propose a machine learning framework for latent circRNA-disease links discovery via a fusion of circRNA sequences and disease ontology. Li et al. [19] use GAT and random walk and restart (RWR) to extract the low-order and high-order neighbor representations from similarity networks of circRNA and disease, respectively. There are two graph auto-encoders (GAE) for circRNA-disease associations prediction, based on integrating these representations.

lncRNAs are antisense RNA molecules with more than 200 nucleotides. They can regulate the transcription and expression of genes and involve in cancer development or suppression, which by specific binding to non-coding regions of target genes [20]. For example, the overexpression of lncRNACTA-929C8 in brain tissue may lead to Alzheimer’s disease, that its high expression is about 1000 times that of other normal tissues [21]. Wang et al. [22] design a weighted matrix factorization method to infer disease-associated lncRNAs. To be specific, the algorithm assigns initial weights to the inter-association and intra-association matrices within the network. It then collaboratively decomposes these matrices into low-rank equivalents, aiming to uncover the inherent relationships among the nodes. Zhang et al. [23] propose a multi-feature coding approach to build the characteristic of linkage among lncRNA and disease samples by combining the six similarity characteristics, and develop an attention CNN to infer possible association between lncRNA and disease. Wu et al. [24] utilize GAE to extract low-dimensional representations of vertices and random forest (RF) to identify the possible relationships between lncRNA and disease. Zhao et al. [25] utilize the GAT to learn vertex representations based on homogeneous and heterogeneous subgraphs. To obtain more semantic information, they perform an attention mechanism for assigning weights to numerous metapath-based subgraphs. For final prediction task, they use neural inductive matrix completion (NIMC) to rebuild the linkages among lncRNA and disease.

Although there have been many efforts to analyze the underlying associations between various ncRNAs and diseases, there are still some challenges [17, 26, 27]: (1) High false-negative association; (2) Insufficient utilization of multi-source information; (3) The noise both from multi-source information and multi-stage methods; (4) The robustness and generalization of the methods are insufficient.

Firstly, as we all know, the traditional wet experiments consume a lot of resources, but are also inefficient and susceptible to the outside world. At present, plentiful computational methods critically depend on the associations between ncRNAs and diseases verified by wet experiments. Unfortunately, there is a phenomenon where the existing open ncRNA-disease databases use 1 and 0 to indicate whether has relationship between them, with very few “1” values pointing a known association and very numerous “0” values pointing an unknown association rather than no association. This phenomenon we called false-negative, and there are many false-negative associations in ncRNA-disease databases, which will impact the performance and interpretability of computational methods [17, 28]. Secondly, abundant previous works enhance performance of methods by fusing the similarity networks of ncRNAs and diseases by a simple average or linear weighting strategy. Therefore, those works ignore that multi-source information may have different contributions to the same prediction task [26, 29]. Thirdly, there are many works using a multi-stage method to integrate multi-source information to improve the performance, and some of those methods also rely on hand-crafted intermediate results. Moreover, noise is contained in most of the similarity information [10, 26]. These will affect the effectiveness and interpretability of methods. Finally, great works focus on two specific bio-entities of interest (e.g., lncRNA and disease), which may lead to a model not being able to get a good result on different datasets when this model uses the same set of parameters. Therefore, the robustness and generalization of methods have to be improved. In conclusion, it is worth noting that reducing false-negatives in original association data, making full and reasonable use of multiple sources of information from bio-entities, and differentiating the significance of various sources of information can enhance the predictive capability of ncRNA-disease associations. Furthermore, it is vital to improve the robustness and generalization of methods. However, there is no complete and effective end-to-end framework to address these challenges.

In this work, to overcome the challenges, we design the GDCL-NcDA that uses the $\textbf{G}$raph learning models and $\textbf{D}$eep matrix factorization based on $\textbf{C}$ontrastive $\textbf{L}$earning for $\textbf{Nc}$RNA-$\textbf{D}$isease $\textbf{A}$ssociations identification. It is an end-to-end computational framework, for integrating divers multi-source information on different HNs. Different from our previous work MHDMF [28], GDCL-NcDA introduces a deep graph learning (deep graph convolutional network-GCNII [30]), employs multiple attention mechanisms, including graph attention network (GAT) and multi-channel attention to enhance the characteristics of within and between similarity networks. GDCL-NcDA also uses DMF to identify potential associations while further adding contrastive learning (CL), which makes the GDCL-NcDA framework have better generalization and robustness. In addition, we perform GDCL-NcDA on more multi-source heterogeneous networks (MHNs) which contain more bio-entities. The GDCL-NcDA has the following advantages:

1. We design an end-to-end computational framework GDCL-NcDA, which is the first to introduce GCNII to fuse the multi-source information of different ncRNAs and diseases based on three different multi-layer heterogeneous networks. Furthermore, GDCL-NcDA is the first to use CL in a chain framework. These multi-layer heterogeneous networks include miRNAs, circRNA, lncRNA, genes, and diseases. GDCL-NcDA consists of four parts: (1) constructing multiple MHNs of ncRNAs and diseases, (2) reconstructing diverse association graphs, (3) establishing various predicted association graphs, (4) generating contrastive loss on reconstructed graphs and predicted graphs.
2. GDCL-NcDA efficiently integrates GCNII, multiple attention mechanisms, DMF, and CL into an end-to-end framework for identifying underlying associations. GDCL-NcDA reduces the false-negative associations via multi-source GCNII and multiple attention mechanisms, which is used to reconstruct the ncRNA-disease association graphs. GDCL-NcDA introduces DMF to take both explicit and implicit feedback into consideration for generating ncRNA-disease associations predictive graphs based on reformulated association graphs. In addition, GDCL-NcDA further utilizes CL to improve generalization and robustness by generating a contrastive loss on the reconstructed graphs and predictive graphs.
3. To assess the capability of GDCL-NcDA, we compare it with seven state-of-the-art methods under 5-fold cross-validation (5CV) and 10-fold cross-validation (10CV) on three different MHNs, and GDCL-NcDA achieves first-rank results. It is shown that GDCL-NcDA can easily extend on different datasets and have better generalization and robustness. Then, we implement ablation experiments to prove the effectiveness of each part and different MHNs, and parameter analysis of GDCL-NcDA to illustrate the choice of parameters. Finally, case studies are performed on miRNA, circRNA, lncRNA and their two corresponding diseases.

### Multi-source heterogenous networks

#### miRNA-gene-disease associations

For miRNA-disease, the positive set of miRNA-disease associations is downloaded from the Human MicroRNA Disease Database (HMDD v2.0) [31]. The miRNA-gene associations are downloaded from the miRWalk2.0 database [32]. The disease-gene associations are downloaded from DisGeNET [33]. We intersect the datasets to remove genes that have no relation with diseases and miRNAs. Meanwhile, we also download the semantic trees of diseases from the U.S. National Library of Medicine (MeSH) [34]. We filter out miRNA-disease associations that their corresponding names are absent in the MeSH descriptors or miRBase records. Then, we get 4266 associations between 285 miRNAs and 197 diseases, and 1789 genes associate with miRNA and disease, respectively.

#### circRNA-gene-disease associations

For circRNA-disease, we download the positive associations of circRNA-disease from CircR2Disease database [35], the circRNA-gene associations from http://cssb2.biology.gatech.edu/knowgene/search.html, and the disease-gene associations from http://cssb2.biology.gatech.edu/knowgene/. We move out diseases and circRNAs that their corresponding names are absent in the MeSH descriptors or the records in Circinteractome and circbank databases. After filtering, there are 418 genes linked with 515 circRNAs and 61 genes linked with 82 diseases, and 563 associations between circRNAs and diseases.

#### lncRNA-gene-disease associations

For lncRNA-disease, we obtain the lncRNA-disease positive linkages from LncRNADdisease database [36], the lncRNA-gene linkages from lncReg database [37], and the disease-gene linkage from DisGeNet database. After removing the duplicate and missing data, we collect 577 linkages among 276 lncRNAs and 125 diseases with 3043 linked genes.

#### Multi-source information

We integrate multi-source information to build three different types of ncRNA-disease MHNs. The MHNs includes the hamming profile, sequence, and gaussian interaction profile kernel (GIPK) similarity of three types of ncRNAs, the hamming profile, semantic, and GIPK similarity of diseases, as well as experimentally valid miRNA-disease, circRNA-disease, lncRNA-disease, miRNA-gene, circRNA-gene, lncRNA-gene, and disease-gene associations. In this work, all the similarity networks of ncRNAs and diseases are treated as graphs with edge weighted. The association matrixes of ncRNA-gene and disease-gene are treated as features for edge-weighted graphs of ncRNAs and diseases, respectively. All the similarity calculations are given in the Supplementary Material.

### Hamming profile similarity

Hamming profile can be used to measure the similarity of a pair of vectors by counting the number of different corresponding elements of the two vectors [38]. According to the biological assumption that similar ncRNAs are always linked with similar diseases, we treat Hamming profile similarity as topological information from the known associations among ncRNAs and diseases. The higher Hamming profile value, the lower similarity in ncRNAs or disease. For diseases, the Hamming profile similarity kernel DHS$(d_{i}, d_{j})$ is defined as follows:

1 $$\begin{aligned} DHS(d_{i}, d_{j}) = \frac{|\textbf{m}(d_{i}) != \textbf{m}(d_{j})|}{|\textbf{m}(d_{i})|} \end{aligned}$$

where $\textbf{m}(d_{i}), \textbf{m}(d_{j})$ represent binary vectors of diseases $d_{i}, d_{j}$, which correspond to the $i^{th}, j^{th}$ column in the ncRNA-disease association matrix $\textbf{M}$.

For ncRNAs, the Hamming profile similarity kernel NHS$(nc_{i}, nc_{j})$ is defined as follows:

2 $$\begin{aligned} MHS(nc_{i}, nc_{j}) = \frac{|\textbf{m}(nc_{i}) !=\textbf{m}(nc_{j})|}{|\textbf{m}(nc_{i})|} \end{aligned}$$

where $\textbf{m}(nc_{i}), \textbf{m}(nc_{j})$ are binary vectors of ncRNAs $nc_{i}, nc_{j}$, which correspond to the $i^{th}, j^{th}$ row in the association matrix $\textbf{M}$.

### Gaussian interaction profile kernel similarity

Gaussian interaction profile kernel (GIPK) can capture topological features of the interaction network of biological entity pairs. The similar bio-entities can be better clustered in a space that describes GIPK similarity. Therefore, the GIPK is a reasonable method for measuring the similarity of bio-entities, and it is widely used. Here, GIPK similarity for diseases $DGS(d_{i}, d_{j})$ between disease $d_{i}$ and $d_{j}$ can be defined as follows:

3 $$\begin{aligned} DGS\left( d_{i}, d_{j}\right) = \exp \left( -\beta _{d}\left\| \textbf{m}\left( d_{i}\right) - \textbf{m}\left( d_{j}\right) \right\| ^{2}\right) \end{aligned}$$

$\beta _{d}$ is a regulation parameter for controlling the kernel bandwidth.

4 $$\begin{aligned} \beta _{d}=\left( \frac{1}{N_{d}} \sum \limits _{i=1}^{N_{d}}\left\| \textbf{m}\left( d_{i}\right) \right\| ^{2}\right) \end{aligned}$$

where $N_{d}$ is the number of all diseases.

Similarly, the GIPK similarity for ncRNAs $NGS\left( nc_{i}, nc_{j}\right)$ between ncRNAs $nc_{i}, nc_{j}$ can be obtained as follows:

5 $$\begin{aligned} \begin{aligned} MGS\left( nc_{i}, nc_{j}\right) = \exp \left( -\beta _{nc}\left\| \textbf{m}\left( nc_{i}\right) - \textbf{m}\left( nc_{j}\right) \right\| ^{2}\right) \end{aligned} \end{aligned}$$

6 $$\begin{aligned} \beta _{nc}=\left( \frac{1}{N_{nc}} \sum \limits _{i=1}^{N_{nc}}\left\| \textbf{m}\left( nc_{i}\right) \right\| ^{2}\right) \end{aligned}$$

where $N_{nc}$ is the number of all ncRNAs.

### Disease semantic similarity

In the last decade, the effectiveness of disease semantic similarity based on Wang et al. [39] has been proved by many previous works, and it is widely used for identifying latent associations between ncRNAs and diseases. In the MeSH disease descriptors, the associations in different diseases can be described as their corresponding Directed Acyclic Graph (DAG) structures. Each node in DAG is a disease and each directed edge is their association. The more similar diseases are, the more common parts of DAGs they share. We obtain the disease semantic similarity $DSS1(d_{i}, d_{j})$ between disease $d_{i}$ and $d_{j}$ by Eq. (7) as follows:

7 $$\begin{aligned} DSS1\left( d_{i}, d_{j}\right) = \frac{\sum _{t \in N\left( d_{i}\right) \cap N\left( d_{j}\right) }\left( C 1_{d_{i}}\left( t\right) +C 1_{d_{j}}\left( t\right) \right) }{\sum _{t \in N\left( d_{i}\right) } C 1_{d_{i}}\left( t\right) +\sum _{t \in N\left( d_{j}\right) } C 1_{d_{j}}\left( t\right) } \end{aligned}$$

where $N\left( d_{i}\right)$ represents a node set on the DAG of disease $d_{i}$. $C 1_{d_{i}}\left( t\right)$ represents the semantic contribution value of a node $t \in N\left( d_{i}\right)$, which is associated with $d_{i}$. For $d_{i}$ itself, $C 1_{d_{i}}\left( t\right) = 1$. For *t* to $d_{i}$, $C 1_{d_{i}}\left( t\right) = \max \left\{ 0.5 * C 1_{d_{i}}\left( t^{\prime }\right) \mid t^{\prime } \in \text{ children } \text{ of } t\right\}$ will increase as their distance decreases.

If a disease occurs in different DAGs, it is a common, and vice versa. The above method for calculating semantic similarity treats every different disease in the same layer as having the same semantic contribution. However, the semantic contribution values of uncommon diseases should be higher than the common diseases [40]. According to previous work [41], we distinguish the semantic contribution values of uncommon diseases by Eq. (8) as follows:

8 $$\begin{aligned} DSS 2\left( d_{i}, d_{j}\right) = \frac{\sum _{t \in N\left( d_{i}\right) \cap N\left( d_{j}\right) }\left( C 2_{d_{i}}\left( t\right) +C 2_{d_{j}}\left( t\right) \right) }{\sum _{t \in N\left( d_{i}\right) } C 2_{d_{i}}\left( t\right) +\sum _{t \in N\left( d_{j}\right) } C 2_{d_{j}}\left( t\right) } \end{aligned}$$

where $C 2_{d_{i}}\left( t\right)$ is the semantic contribution value of *t* to $d_{i}$ can be defined as Eq. (9):

9 $$\begin{aligned} C 2_{d_{i}}\left( t\right) = -\log \left( \frac{ \text{ the } \text{ number } \text{ of } \text{ DAGs } \text{ including } t}{ \text{ the } \text{ number } \text{ of } \text{ diseases } }\right) \end{aligned}$$

Inspired by previous work [41], we calculate the final disease semantic similarity $DSS(d_{i}, d_{j})$ between disease $d_{i}$ and $d_{j}$, which integrating the results of the above two semantic similarity calculations and describing as below:

10 $$\begin{aligned} DSS\left( d_{i}, d_{j}\right) = \frac{DSS 1\left( d_{i}, d_{j}\right) +DSS 2\left( d_{i}, d_{j}\right) }{2} \end{aligned}$$

### ncRNA sequence similarity

To make use of the ncRNA sequence information, we compute the ncRNA sequence similarity scores $NSS\left( nc_{i}, nc_{j}\right)$ based on Smith-Waterman (SW) [42] method. This sequence pairwise alignment method is packaged using the Biopython, a python tool. The sequence information of miRNAs, circRNAs, and lncRNAs is downloaded from miRBase [34] database, CircInteractome [43] database and circBank [44] database, and LncRNADisease [36] database, respectively. In this work, *NSS* represents the ncRNA sequence similarity network. The weight of each edge in *NSS* needs to be normalized to the range [0,1] as follows:

11 $$\begin{aligned} \begin{aligned} NSS\left( nc_{i}, nc_{j}\right) = \frac{SW\left( nc_{i}, nc_{j}\right) }{max(SW(nc_{i}, nc_{i}), SW(nc_{j}, nc_{j}))} \end{aligned} \end{aligned}$$

where $NSS\left( nc_{i}, nc_{j}\right)$ denotes the Smith-Waterman score between ncRNA $nc_{i}$ and $nc_{j}$.

## Methods

### Model framework

We design a widely effective computational framework GDCL-NcDA for identifying latent different types of ncRNA-disease associations. In effect, the more different varieties of data there are, the more complementary information there is. Many previous works have shown that exploiting multi-source information does help computational methods improve their performance. In this work, our end-to-end framework utilize multi-source information from three large MHNs to reduce the influence of the false-negative associations and relieve the noise which may be introduced by a multi-stage method.

**Fig. 1:**
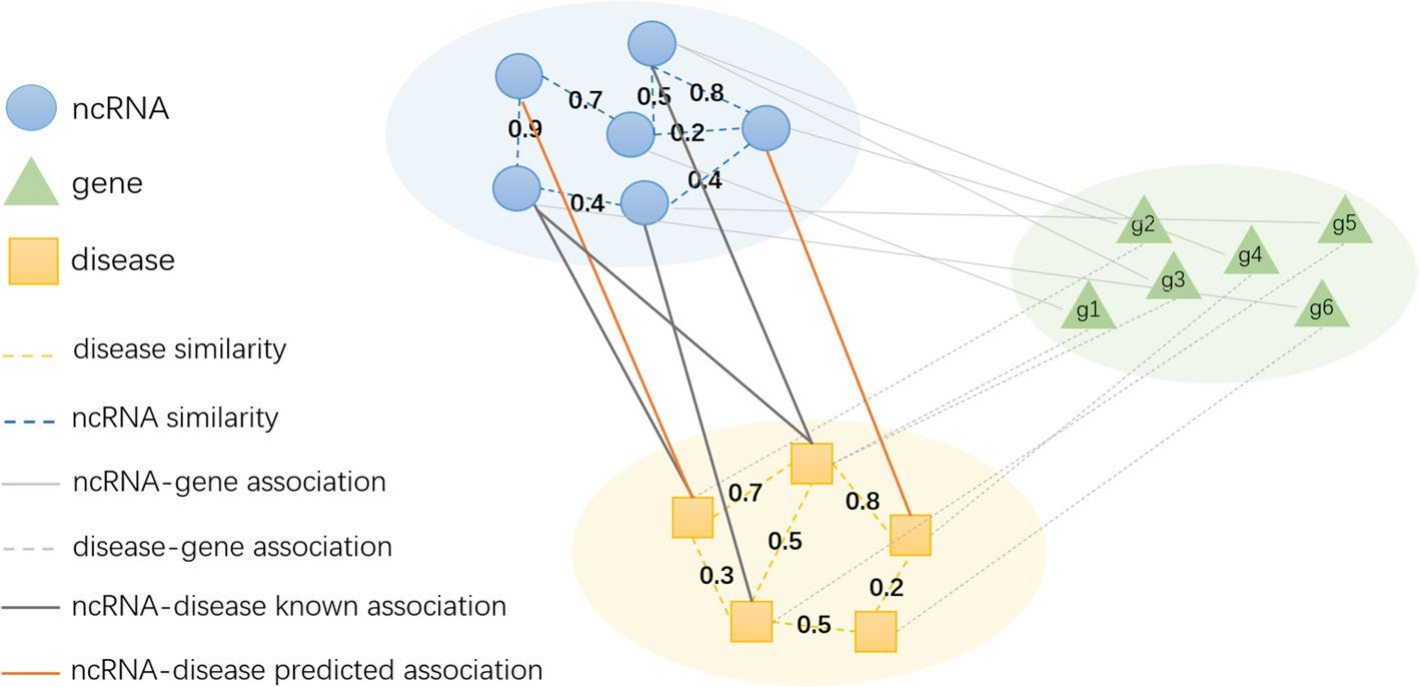
The construction of multi-source heterogeneous network (MHN) of ncRNA-gene-disease

**Fig. 2:**
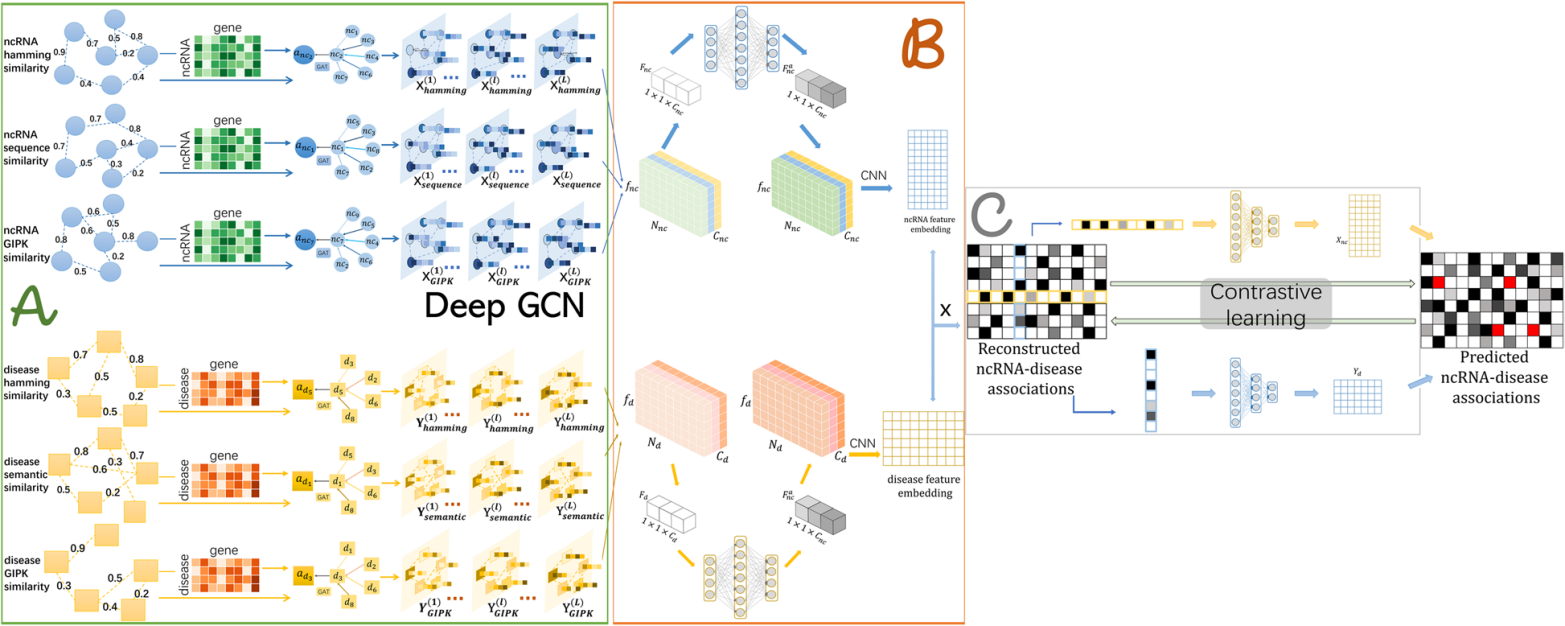
An illustration of the GDCL-NcDA framework. $\textbf{A}$ The multi-source deep graph learning is to obtain significance within similarity network and encode every similarity network. $\textbf{B}$ The multichannel attention mechanism is performed to obtain significance among diverse similarity networks. The reconstruction of association graph (matrix) for downstream predictive task. $\textbf{C}$ The DMF for final identification task based on reformulated association score matrix. The contrastive loss generated on the reconstructed graph and predicted graph

Figures 1 and 2 show the overall flow of GDCL-NcDA, which is constitutive of four parts: (1) constructing multiple MHNs of ncRNAs and diseases (Fig. 1), (2) reconstructing association graphs (matrixes) (Fig. 2. *A* and *B*), (3) establishing predicted association graphs (matrixes) (Fig. 2. *C*), (4) generating contrastive loss on reconstructed graphs and predicted graphs (Fig. 2. *C*). For constructing multiple MHNs of ncRNAs and diseases, we construct three multiple layers MHNs including similarity profiles and interaction profiles of miRNAs, circRNA, lncRNA, genes, and diseases. For reconstructing association graphs, we use GAT to reduce the the impact of noise in the similarity networks and enhance the characteristics within the similarity network. GCNII is used to encode the different similarity profiles and interaction profiles, and channel attention mechanism to enhance the characteristics between the similarity networks. For establishing predicted association graphs, we employ DMF to predict the latent associations based on reconstructed association graphs. Furthermore, we introduce a novel contrastive optimization module to generate a collaborative contrastive loss of reconstructed association graphs and predicted graphs.

### Graph attention mechanism

Graph attention network (GAT) [45] is a novel convolution-style neural network. It is a valid method for graph representation learning, which can solve the weaknesses of previous graph convolution-based approaches. In GAT, the nodes can take part in their neighborhoods’ features. In different sized neighborhoods, GAT is capable of implicitly assigning different significances to different nodes. In this work, we use GAT to capture the characteristics within multiple homogeneous similarity networks of ncRNA and disease.

In GDCL-NcDA, GAT is adopted to obtain the shallower embeddings on each similarity networks ncRNA and disease for downstream works, which can reduce the effect of noise in the similarity networks. We can obtain the attention-based similarity networks after GAT. We use GAT to enhance the characteristics within each similarity network. GAT utilizes a masked self-attention mechanism to learn the significance of its neighbors first. More specifically, it apples linear transformation on nodes *i*, *j* (node pointing a disease here) in a similarity graph $\mathcal {G}$ and employs self-attention on the nodes by a shared attentional mechanism, a mapping function $f_{a}(\cdot )$, which can calculate attention coefficient $w_{ij}^{gat}$ as follows:

12 $$\begin{aligned} w_{i j}^{gat} = f_{a}(\textbf{W}_{gat}\textbf{f}_{i}, \textbf{W}_{gat}\textbf{f}_{j}) ; \end{aligned}$$

where $\textbf{F}_{d} = \{\textbf{f}_{1}, \textbf{f}_{2}, \cdots , \textbf{f}_{N_{d}}\}, \textbf{f}_{i}, \textbf{f}_{j} \in \mathbb {R}^{F_{d}}$ is the input feature of disease nodes, where $N_{d}$ is the number of disease nodes, and $F_{d}$ is the dimensionality of each node, and $\textbf{W}_{gat} \in \mathbb {R}^{F_{d} \times N_{d}}$. The GAT output of disease $\textbf{F}^{'}_{d} = \{\mathbf {f^{'}}_{1}, \mathbf {f^{'}}_{2}, \cdots , \mathbf {f^{'}}_{N_{d}}\}, \mathbf {f^{'}}_{i} \in \mathbb {R}^{N_{d}}$

In this model, each node can participate in each other node, without all structural information. By introducing the masked attention, the $w_{ij}^{gat}$ for nodes $j \in \mathcal {N}_{i}$, where $\mathcal {N}_{i}$ denotes $1^{st}$-order neighbors of node *i* in the $\mathcal {G}$. To make the coefficients easy to compare between different nodes, we normalize the significance of different neighbor nodes by softmax function can be expressed as follows:

13 $$\begin{aligned} \alpha _{ij} = softmax_{j}(w_{ij}^{gat}) = \frac{exp(w_{ij}^{gat})}{\sum _{k \in \mathcal {N}_{i}}exp(w_{ik}^{gat})} \end{aligned}$$

In this work, we apply the LeakyReLU nonlinearity, by fully expanding out, the coefficients calculated by the attention mechanism can be formulated as follows:

14 $$\begin{aligned} \alpha _{ij} = \frac{exp(LeakyReLU ({\textbf{a}}^{T}[\textbf{W}_{gat}\textbf{f}_{i}||\textbf{W}_{gat}\textbf{f}_{j}]))}{\sum _{k \in \mathcal {N}_{i}}exp(LeakyReLU ({\textbf{a}}^{T}[\textbf{W}_{gat}\textbf{f}_{i}||\textbf{W}_{gat}\textbf{f}_{k}]))} \end{aligned}$$

where ${\textbf{a}} \in \mathbb {R}^{2N_{d}}$ is a weight vector to parameterize the attention layer. $\cdot ^{T}$ represents matrix transposition and $\Vert$ represents the concatenation operation.

Subsequently, we can obtain the aggregated features of each node that linearly combines the normalized attention coefficients and nodes features. The aggregated features use a potentially nonlinear activation function $\sigma (\cdot )$ to be the final node features. Then, the formation of GAT output $\mathbf {f^{'}}_{i}$ is shown as follows:

15 $$\begin{aligned} \mathbf {f^{'}}_{i} = \sigma \left(\sum \limits _{j \in \mathcal {N}_{i}} \alpha _{ij} \textbf{W}_{gat}\textbf{f}_{j}\right) \end{aligned}$$

In this work, to reduce the impact of self-attention and stabilize the learning process of nodes importance, we further employ multi-head attention. Specifically, we concatenate the node features which executing *K* independent self-attention, then the Eq. (15) can be rewritten as follows:

16 $$\begin{aligned} \mathbf {f^{'}}_{i} = \Vert _{k=1}^K \sigma \left( \sum \limits _{j \in \mathcal {N}_{i}} \alpha _{i j}^{k} \textbf{W}_{gat}^{k} \textbf{f}_{j}\right) . \end{aligned}$$

where $\Vert$ denotes the concatenation operation, $\alpha _{i j}^{k}$ denote the normalized attention coefficients calculated by $k^{th}$ self-attention $(a_{gat}^{k})$, and $\textbf{W}_{gat}^{k}$ denotes the corresponding weight matrix. Correspondingly, we can obtain the final GAT output of disease $\textbf{F}^{'}_{d} \in \mathbb {R}^{N_{d} \times N_{d}}$, as well as ncRNA $\textbf{F}^{'}_{nc} \in \mathbb {R}^{N_{nc} \times N_{nc}}$, $N_{nc}$ is the number of ncRNA nodes. We treat these GAT outputs as attention-adjacency matrixes of ncRNA $\textbf{A}_{nc}$ and disease $\textbf{A}_{d}$ for the downstream reconstruction task, which also called attention-based similarity networks of ncRNAs $\mathcal {G}^{a}_{nc}$ and diseases $\mathcal {G}^{a}_{d}$.

### Deep graph convolution network

Graph convolution network (GCN) and its variants are vital components of graph learning, which can obtain the low-dimensional vector embedding of nodes [46]. Despite they show excellent performance in varieties of application areas on real-world datasets, most of the recent models are shallow, such as GCN [47] and GAT [45], to accomplish their perfect performance with 2-layer models. Stacking more graph convolution layers and adding non-linearity can cause a phenomenon, called *over-smoothing*, which tends to impact these models’ performance. Chen et al. [30] develop the GCNII to effectively relieve the problem of *over-smoothing* by using Initial residual and Identity mapping techniques. In this work, we utilize the GCNII for similarity-specific learning, where a GCNII is trained for each attention-based similarity network to apply the association graph reformulation component.

In GDCL-NcDA, we treat every attention-based similarity network as a edge-weighted graph $\mathcal {G}^{a}_{nc} = (\mathcal {V}_{nc}, \mathcal {E}_{nc})$ and $\mathcal {G}^{a}_{d} = (\mathcal {V}_{d}, \mathcal {E}_{d})$. There are two inputs for a GCNII model: (1) attention-adjacency matrixes $\textbf{A}_{nc} \in \mathbb {R}^{N_{nc} \times N_{nc}}$ and $\textbf{A}_{d} \in \mathbb {R}^{N_{d} \times N_{d}}$ representing the graph structure description, where $N_{nc}$ is the number of ncRNAs and $N_{d}$ is the number of diseases; (2) nodes feature matrixes $\textbf{X} \in \mathbb {R}^{N_{nc} \times F_{nc}}$ and $\textbf{Y} \in \mathbb {R}^{N_{d} \times F_{d}}$, where $F_{nc}$ and $F_{d}$ are the feature dimensionality of ncRNAs and diseases, respectively. We treat ncRNA-gene and disease-gene as the feature matrixes of ncRNA-ncRNA edge-weighted graphs and disease-disease edge-weighted graphs, respectively. Each attention-based similarity network trained by one GCNII, the GCNII can be built by stacking multiple convolutional layers, for ncRNA, the embedding of the $l^{th}, l = \{1, 2, \cdots , L\}$ layer defined as follows:

17 $$\begin{aligned} \textbf{X}^{(l+1)}=\delta \left(\left( 1-\alpha _{l}\right) \tilde{\textbf{P}} \textbf{X}^{(l)}+\alpha _{l} \textbf{X}) ((1-\beta _{l}) \textbf{I}_{n}+\beta _{l} \textbf{W}_{gcnii}^{(l)}\right) \end{aligned}$$

For disease, the embedding of the $l^{th}$ layer can be written as follows:

18 $$\begin{aligned} \textbf{Y}^{(l+1)}=\delta \left(\left( 1-\alpha _{l}\right) \tilde{\textbf{P}} \textbf{Y}^{(l)}+\alpha _{l} \textbf{Y}\right) \left((1-\beta _{l}) \textbf{I}_{n}+\beta _{l} \textbf{W}_{gcnii}^{(l)}\right) \end{aligned}$$

where $\alpha _{l}$ and $\beta _{l}$ are hyperparameters. We need ensure that the final embedding of every node retains a fraction of $\alpha _{l}$ from input feature if the layers stacked, $\alpha _{l} = 0.2$ we set here. Setting $\beta _{l}$ is to ensure the decay of the weight matrix adaptively increases as more layers stacked, in here $\beta _{l} = log(\lambda / l) \approx \lambda / l$, where $\lambda$ is a hyperparameter.

$$\begin{aligned} \tilde{\textbf{P}} = \tilde{\textbf{D}}^{-1/2}\tilde{\textbf{A}}\tilde{\textbf{D}}^{-1/2} = (\textbf{D} + \textbf{I}_{n})^{-1/2} (\textbf{A} + \textbf{I}_{n})(\textbf{D} + \textbf{I}_{n})^{-1/2}, \end{aligned}$$

which is the graph convolution matrix with the renormalization trick, where $\textbf{D}$ is the diagonal degree matrix of $\textbf{A}$. $\textbf{I}_{n}$ is identity mapping. We can obtain the final deep graph learning embeddings of ncRNA $\textbf{E}^{X} \in \mathbb {R}^{N_{nc} \times f_{nc}}$ and disease $\textbf{E}^{Y} \in \mathbb {R}^{N_{d} \times f_{d}}$ from multiple source information, $f_{nc}$ and $f_{d}$ are the dimensionality of embeddings.

### Multi-channel attention mechanism

Many previous works normally use a simple average or a linear weighting strategy to integrate the multiple similarity information, which ignores the difference in contribution of different source similarity information [48]. In this work, we perform the multi-channel attention mechanism to capture the characteristics between the multiple similarity networks of ncRNA and disease.

From Fig. 2. C, the embedding tensor $\mathscr {T}$ is stacked by all similarity embedding matrixes from the upper deep multi-source information graph learning, and each embedding matrixes are treated as a channel for an attention layer. Then, we model the significance of each channel (similarity) to increase or decrease the contribution of diverse source similarities. $C_{nc}, C_{d}$ are the numbers of channels from ncRNA and disease, respectively. By squeezing embedding tensors of ncRNA $\mathscr {T}_{X} = [\textbf{E}_{1}^{X}, \textbf{E}_{2}^{X}, \cdots , \textbf{E}_{C_{nc}}^{X}], \mathscr {T}_{X} \in \mathbb {R}^{N_{nc} \times f_{nc} \times C_{nc}}$ and disease $\mathscr {T}_{Y} = [\textbf{E}_{1}^{Y}, \textbf{E}_{2}^{Y}, \cdots , \textbf{E}_{C_{d}}^{Y}], \mathscr {T}_{Y} \in \mathbb {R}^{N_{d} \times f_{d} \times C_{d}}$. We can get the one-dimensional (1D) features of ncRNA $\mathscr {F}_{X} \in \mathbb {R}^{1 \times 1 \times C_{nc}}$ and disease $\mathscr {F}_{Y} \in \mathbb {R}^{1 \times 1 \times C_{d}}$. Specifically, for the $c^{th}_{nc}$, $c^{th}_{d}$ embedding matrix of ncRNA $\textbf{E}_{c_{nc}}^{X}$ and disease $\textbf{E}_{c_{d}}^{Y}$, the values $f_{c_{nc}}, f_{c_{d}}$ in $\mathscr {F}_{X}, \mathscr {F}_{Y}$ are calculated as follows:

19 $$\begin{aligned} f_{c_{nc}}=\Theta _{squeez}\left( \textbf{E}_{c_{nc}}^{X}\right) =\frac{\sum _{i=1}^{f_{nc}} \sum _{j=1}^{N_{nc}} \textbf{E}_{c_{nc}}^{X}(i, j)}{f_{nc} \times N_{nc}} \end{aligned}$$

20 $$\begin{aligned} f_{c_{d}}=\Theta _{squeez}\left( \textbf{E}_{c_{d}}^{Y}\right) =\frac{\sum _{i=1}^{f_{d}} \sum _{j=1}^{N_{d}} \textbf{E}_{c_{d}}^{Y}(i, j)}{f_{d} \times N_{d}} \end{aligned}$$

We capture the significance of channels is computed as attention weights by using attention mechanism:

21 $$\begin{aligned} \mathscr {F}_{X}^{a}= & {} \Theta _{a t t e n t i o n}\left( \mathscr {F}_{X}, \textbf{W}^{X}\right) \nonumber \\= & {} Sigmoid\left( \textbf{W}_{2}^{X} \cdot Relu\left( \textbf{W}_{1}^{X} \mathscr {F}_{X}\right) \right) \nonumber \\= & {} \left[ f_{1}^{a}, f_{2}^{a}, \dots , f_{C_{nc}}^{a} \right] \end{aligned}$$

22 $$\begin{aligned} \mathscr {F}_{Y}^{a}= & {} \Theta _{a t t e n t i o n}\left( \mathscr {F}_{Y}, \textbf{W}^{Y}\right) \nonumber \\= & {} Sigmoid\left( \textbf{W}_{2}^{Y} \cdot Relu\left( \textbf{W}_{1}^{Y} \mathscr {F}_{Y}\right) \right) \nonumber \\= & {} \left[ f_{1}^{a}, f_{2}^{a}, \dots , f_{C_{d}}^{a} \right] \end{aligned}$$

where $\textbf{W} = \{\textbf{W}_{1}, \textbf{W}_{2}\}$ is the training parameter, $f_{C_{nc}}^{a}, f_{C_{d}}^{a}$ are values in $\mathscr {F}_{X}^{a} \in \mathbb {R}^{1 \times 1 \times C_{nc}}, \mathscr {F}_{Y}^{a} \in \mathbb {R}^{1 \times 1 \times C_{d}}$, which are attentional 1D features of ncRNA and disease, respectively.

Finally, we obtain the normalized channel embeddings with attention weights as follows:

23 $$\begin{aligned} \tilde{\textbf{E}}_{c_{nc}}^{X}=\Theta _{\text{ weighted }}\left( \textbf{E}_{c_{nc}}^{X}, f_{c_{nc}}^{a}\right) =f_{c_{nc}}^{a} \cdot \textbf{E}_{c_{nc}}^{X} \end{aligned}$$

24 $$\begin{aligned} \tilde{\textbf{E}}_{c_{d}}^{Y}=\Theta _{\text{ weighted }}\left( \textbf{E}_{c_{d}}^{Y}, f_{c_{d}}^{a}\right) =f_{c_{d}}^{a} \cdot \textbf{E}_{c_{d}}^{Y} \end{aligned}$$

as aforementioned, we can get the enhanced channel embeddings of ncRNA $\tilde{\mathscr {T}}_{X} = [\tilde{\textbf{E}}_{1}^{X}, \tilde{\textbf{E}}_{2}^{X} , \dots , \tilde{\textbf{E}}_{C_{nc}}^{X}]$ , and disease $\tilde{\mathscr {T}}_{Y} = [\tilde{\textbf{E}}_{1}^{Y}, \tilde{\textbf{E}}_{2}^{Y} , \dots , \tilde{\textbf{E}}_{C_{d}}^{Y}]$.

### The association graph reconstruction

We employ CNN to generate the final embeddings of ncRNA $\textbf{X}_{nc}^{\prime }$ and disease $\textbf{Y}_{d}^{\prime }$ based on the enhanced multiple channel embeddings, $\textbf{X}_{nc}^{\prime }$ and $\textbf{Y}_{d}^{\prime }$ are represented as follows:

25 $$\begin{aligned} \textbf{X}_{nc}^{\prime } = stack(\textbf{Xout}_{k}) \end{aligned}$$

26 $$\begin{aligned} \textbf{Xout}_{k} = \Theta _{agg}(\tilde{\mathscr {T}}_{X}) = \textbf{bias}_{k} + \sum \limits _{i = 1}^{C_{nc}} { \tilde{\textbf{E}}_{i}^{X} * \textbf{W}_{k}^{nc}} \end{aligned}$$

27 $$\begin{aligned} \textbf{Y}_{d}^{\prime } = stack(\textbf{Yout}_{k}) \end{aligned}$$

28 $$\begin{aligned} \textbf{Yout}_{k} = \Theta _{agg}(\tilde{\mathscr {T}}_{Y}) = \textbf{bias}_{k} + \sum \limits _{i = 1}^{C_{d}} { \tilde{\textbf{E}}_{i}^{Y} * \textbf{W}_{k}^{d}} \end{aligned}$$

where $\textbf{W}_{k}^{nc} \in \mathbb {R}^{f_{nc} \times 1}$ and $\textbf{W}_{k}^{d} \in \mathbb {R}^{f_{d} \times 1}$, $f_{nc}$ and $f_{d}$ are the numbers of feature from GCNII embeddings.

Then, we reconstruct the ncRNA-disease association graph $\textbf{ReG} \in \mathbb {R}^{N_{nc} \times N_{d}}$ by using Matrix Factorization (MF), which can be described as:

29 $$\begin{aligned} \textbf{ReG} = \textbf{X}_{nc}^{\prime } \cdot {\textbf{Y}_{d}^{\prime }}^{T} \end{aligned}$$

### Deep matrix factorization

Matrix Factorization (MF) is a latent factor model, which performs outstanding capacity in information mining of the recommender tasks [49]. Many previous works utilize MF methods of predicting the linkages between biological entities successfully [3, 50, 51]. As we all know, the associations between biological entities are very sparse, which will affect the performance of the computational methods. In order to alleviate the impact of this problem, many methods add relevant similarity information to assist a prediction task [52]. However, modeling only linear features extracted by MF is insufficient to extract complicated associations between ncRNAs and diseases. Deep matrix factorization (DMF) captures non-linear features between ncRNA and disease, which is based on all explicit and implicit feedback and improves the prediction performance.

There are three steps in this part. Firstly, we extract the row vector and column vector of the reconstructed associations $\textbf{ReG}$ as the original features of ncRNA $\textbf{ReG}_{i*}$ and disease $\textbf{ReG}_{*j}$, respectively. $\textbf{ReG}_{i*}$ and $\textbf{ReG}_{*j}$ contain the association patterns of ncRNA $nc_{i}$ and disease $d_{j}$, and considered as associations between $i^{th}$ ncRNA and all diseases, as well as $j^{th}$ disease and all ncRNAs, respectively. There is a high false-negative in the original ncRNA-disease association $\textbf{M}$, because that 1 is known link with experimental backing (explicit feedback), while 0 is unknown link rather than no link (implicit feedback). We obtain predicted scores for some unknown relations in $\textbf{ReG}$ to reduce the false-negative. Meanwhile, we retain the original “1” values in ncRNA-disease associations. The implicit feedback is denoted by non-zero values between 0 and 1, rather than 0 only. We further perform implicit feedback composed of association patterns to enhance performance. Secondly, we treat $\textbf{ReG}_{i*}$ and $\textbf{ReG}_{*j}$ as inputs of multiple fully connected layers, projecting ncRNA and disease into potential structured space. To be more specifically, we generate the feature of ncRNA $\textbf{x}_{i}$ (as same as the feature of disease $\textbf{y}_{j}$) from this process is as follows:

30 $$\begin{aligned} h_{1}= & {} \textbf{W}^{\prime }_{1^{\prime }} \textbf{ReG}_{i *} \nonumber \\ h_{l}= & {} f_{\theta }\left( \textbf{W}^{\prime }_{l^{\prime }-1} l^{\prime }_{l^{\prime }-1}+\textbf{b}_{l^{\prime }}\right) , l^{\prime }=2, \ldots , L^{\prime }-1 \nonumber \\ x_{i}= & {} f_{\theta }\left( \textbf{W}^{\prime }_{L^{\prime }} h_{L^{\prime }-1}+\textbf{b}_{L^{\prime }}\right) \end{aligned}$$

where $h_{l^{\prime }} (l^{\prime } = 1, \dots , L^{\prime }-1)$ denotes the $l^{\prime th}$ hidden layer and the $L^{\prime }$ denotes the number of hidden layers. $\textbf{W}^{\prime }_{l^{\prime }}$ and $\textbf{b}_{l^{\prime }}$ are the weight matrix and the bias term on the $l^{\prime th}$ hidden layer, respectively. $f_{\theta }(\cdot )$ is a nonlinear activation function, we use the Rectified Linear Unit (ReLU) here.

Thirdly, we obtain the final features of ncNRA $\textbf{X}_{nc} = \{ \textbf{x}_{1}, \textbf{x}_{2}, \dots , \textbf{x}_{m}\}$ and disease $\textbf{Y}_{d} = \{\textbf{y}_{1}, \textbf{y}_{2}, \dots , \textbf{y}_{n}\}$. We can get the final ncRNA-disease association predicted graph $\textbf{PrG} \in \mathbb {R}^{N_{nc} \times N_{d}}$ by MF as below:

31 $$\begin{aligned} \textbf{PrG} = \textbf{X}_{nc} \cdot {\textbf{Y}_{d}}^{T} \end{aligned}$$

the higher value $\textbf{PrG}_{ij}$ is, the more possibility association between ncRNA $nc_{i}$ and disease $d_{j}$, and vice versa.

In GDCL-NcDA, we use mean square error as a loss function. It is which is achieved by minimizing the Frobenius norm of the difference between $\textbf{PrG}$ and $\textbf{M}$. The loss function is given as follows:

32 $$\begin{aligned} Loss_{DMF}=\left\| \textbf{M}-\textbf{PrG}\right\| _{F}^{2} \end{aligned}$$

### Co-contrastive learning

Contrastive Learning (CL) demonstrates excellent ability of unsupervised performance in graph representation learning [53–56]. Initially, Velickovic et al. [53] and Sun et al. [57] learn the expressive representations of graphs or nodes, which by maximizing the interactive information of different graininess among graph-level representations and substructure-level representations. Peng et al. [58] obtain interactive information between input and representations of nodes and edges by performing two discriminators. You et al. [59–61] propose various augmentations for graph-level representation learning.

In this work, we use the CL to learn the interactive information of representations of nodes and edges from reconstructed association graph and predicted association graph, rather than contrasting different augmented views of examples. The purpose of CL used is to improve the generalization ability of our framework and supervise the learning of the latent linkage prediction task. The co-contrastive learning loss $Loss_{CL}$ for each positive pair $(\textbf{reg}_{i}, \textbf{prg}_{i})$ of the reconstructed association graph and predicted association graph can be defined as follows:

33 $$\begin{aligned} Loss_{CL} = -\frac{1}{2N} \sum \limits _{i=1}^{N}[l(\textbf{reg}_{i}, \textbf{prg}_{i}) + l(\textbf{prg}_{i}, \textbf{reg}_{i})] \end{aligned}$$

34 $$\begin{aligned}{} & {} l(\textbf{reg}_{i}, \textbf{prg}_{i}) = \nonumber \\{} & {} log \frac{e^{\phi (\textbf{reg}_{i}, \textbf{prg}_{i})/\mathcal {T}}}{e^{\phi (\textbf{reg}_{i}, \textbf{prg}_{i})/\mathcal {T}} + \sum _{k \ne i} e^{\phi (\textbf{reg}_{i}, \textbf{prg}_{k})/\mathcal {T}} + \sum _{k \ne i} e^{\phi (\textbf{reg}_{i}, \textbf{reg}_{k})/\mathcal {T}}} \end{aligned}$$

where $\textbf{reg}_{i}$ is the embedding of a node $\textbf{reg}_{i}$ in $\textbf{ReG}$ treated as the anchor, and $\textbf{prg}_{i}$ is the embedding in $\textbf{PrG}$, which is the positive sample. We treat the embeddings of other nodes in both graphs as negatives (positives and negatives mean that have relations and no relations). $\mathcal {T}$ is a augmentation function, the critic $\phi (\textbf{reg}, \textbf{prg}) = sim(g(\textbf{reg}), g(\textbf{prg}))$, where $sim(\cdot )$ is the cos similarity and $g(\cdot )$ is linear projection to enhance the expression power of the critic function [30].

Finally, the optimization objective of our framework consists of three parts: the multi-source graph learning loss, the DMF loss, and the contrastive loss. The final loss function of *Loss* can be shown as follows:

35 $$\begin{aligned} Loss = \left\| \textbf{M}-\textbf{ReG}\right\| _{F}^{2} + \left\| \textbf{M}-\textbf{PrG}\right\| _{F}^{2} + Loss_{CL} \end{aligned}$$

## Experiments

In this section, we implement experiments to implement the following queries: (1) Is it viable and efficient to be a wide method for identifying latent associations among multiple types of ncRNAs and diseases based on the proposed GDCL-NcDA? (2) Is it useful to integrate deep graph learning, DMF and co-contrastive learning into an end-to-end framework? (3) Is it beneficial to use information on larger MHNs?

### Comparison with highly related methods

To prove the viable and efficient of GDCL-NcDA, we compare the GDCL-NcDA framework to another seven advanced methods in recent years. The 5CV and the 10CV are performed to evaluate the performance of GDCL-NcDA and those seven methods on the same MHNs. All known associations between ncRNA and disease are treated as positive samples and unknown associations are treated as candidate samples. In K-fold cross-validation (K is 5 or 10), (step 1) all proved associations are shuffled randomly and divided into K groups; (step 2) for each unique group, it is toke as a test dataset and the remaining groups are toke as training dataset; (step 3) repeat step 2 K times, each time with a different group. Our results are the average of the K group of results for K-fold cross-validation. According to the articles of baselines, the settings of the parameters in these methods are adjusted to the optimal on our datasets. For our GDCL-NcDA, the GCNII layers is set to 5, the CNN feature dimensionality is set to 96, the DMF layers is set to 2, the DMF feature dimensionality is set to 96, the learning rate is set to 0.001, the adaptive moment estimation (Adam) optimizer is used as the optimizer. It is worth noting that our experiments on the three different MHNs are all based on the above set of parameters. We also utilize the area under the receiver operating characteristic curve (AUC) and the area under the precision/recall curve (AUPRC) to assess the performance of those eight methods. All experiments are repeated 10 times to obtain a sound estimate of prediction results.

#### Baselines

$\mathbf {MDA-SKF}$ [11]: A novel diverse similarity kernels integration for miRNA-disease relations prediction. MDA-SKF develops the Similarity Kernel Fusion (SKF) to integrate different similarity kernels of miRNA and disease extracted in two subspaces, respectively, and then, performs the Laplacian regularized least-squares method to predict the potential miRNA-disease relations.

$\textbf{NIMCGCN}$ [62]: Neural Inductive Matrix Completion (NIMC) with GCN for miRNA-disease relationships identification. NIMCGCN is the first model that uses GCN to learn miRNA and disease representations based on their corresponding similarity networks. Then, the learned representations are treated as inputs for a novel NIMC method to obtain a miRNA-disease relationship matrix completion.

$\textbf{MMGCN}$ [26]: A multi-source GCN with attention mechanism for miRNA-disease links prediction. MMGCN learns embeddings of miRNA and disease via GCN encoding their various corresponding similarity views, respectively. It further employs attention mechanism to differentiate the embeddings from different views for prediction task.

$\textbf{DMFCDA}$ [63]: DMF for circRNA-disease linkages inference. DMFCDA employs a projection layer to learn underlying features of circRNA and disease from original linkages between circRNA and diseases only. By modeling the non-linear linkages, it can learn complex information from data and take both explicit and implicit feedback into consideration.

$\textbf{DMFMSF}$ [27]: DMF with SVD and SKF for ncRNA-disease relations discovery. DMFMSF first uses SKF to integrate three similarities of ncNRA and disease, respectively. Then, it extracts linear and non-linear characteristics by Singular Value Decomposition (SVD) and DMF. In finally, it combines linear and non-linear characteristics to discover potential ncRNA-disease relations.

$\mathbf {CKA-HGRTMF}$ [3]: A novel model of three matrixes factorization with hypergraph-regular terms for ncRNA-disease relationship prediction. It assesses the degree of association by the bilateral projection matrix and two potential characteristic matrixes of ncRNA and disease, respectively. It further uses two graph regular terms on ncRNA and disease characteristics to enhance the predict performance.

$\textbf{MHDMF}$ [28]: A multi-source GCN and DMF for miRNA-disease associations identification. MHDMF learns and enhances embeddings of miRNA and disease by GCN and channel attention from their diverse corresponding similarity networks, respectively. At last, it further uses DMF to identify latent associations based on the embeddings.

#### Performance comparison

In Tables 1, 2, 3, and 4, we demonstrate all comparison results to illustrate the feasibility and the effectiveness of GDCL-NcDA. Our framework GDCL-NcDA performs outstanding among these comparison methods. As the comparative results of GDCL-NcDA under 5CV and 10CV have tiny differences, our GDCL-NcDA has better robustness than other methods. More importantly, the GDCL-NcDA framework has stable performance on different MHNs and strong generalization in the face of different datasets.

**Table 1:** AUC of GDCL-NcDA and seven comparison methods under the 5CV

Methods	miRNA	circRNA	lncRNA
MDA-SKF	0.9068	0.9661	0.9222
NIMCGCN	0.8959	0.8891	0.8612
MMGCN	0.9063	0.9578	0.8788
DMFCDA	0.8519	0.8629	0.8044
DMFMSF	0.9247	0.9397	0.9292
CKA-HGRTMF	0.9675	0.9732	0.9185
MHDMF	0.9240	0.9434	0.9314
GDCL-NcDA	**0.9761**	**0.9849**	**0.9382**

**Table 2:** AUPRC of GDCL-NcDA and seven comparison methods under the 5CV

Methods	miRNA	circRNA	lncRNA
MDA-SKF	0.6332	0.7342	0.6074
NIMCGCN	0.8611	0.9094	0.8771
MMGCN	0.9159	0.9622	0.9053
DMFCDA	0.8632	0.8868	0.8228
DMFMSF	0.9366	0.9448	0.9471
CKA-HGRTMF	0.8712	0.9173	0.8017
MHDMF	0.9452	0.9783	0.9537
GDCL-NcDA	**0.9806**	**0.9890**	**0.9515**

**Table 3:** AUC of GDCL-NcDA and seven comparison methods under the 10CV

Methods	miRNA	circRNA	lncRNA
MDA-SKF	0.9291	0.9821	0.9375
NIMCGCN	0.9187	0.9169	0.8992
MMGCN	0.9097	0.9595	0.8990
DMFCDA	0.8726	0.8567	0.8163
DMFMSF	0.9265	0.8245	0.8743
CKA-HGRTMF	0.9274	0.9173	0.9226
MHDMF	0.9611	0.9087	0.9339
GDCL-NcDA	**0.9807**	**0.9823**	**0.9436**

**Table 4:** AUPRC of GDCL-NcDA and seven comparison methods under the 10CV

Methods	miRNA	circRNA	lncRNA
MDA-SKF	0.6404	0.7349	0.6108
NIMCGCN	0.9388	0.9231	0.8997
MMGCN	0.9160	0.9601	0.9157
DMFCDA	0.8913	0.8919	0.8363
DMFMSF	0.9296	0.8855	0.8134
CKA-HGRTMF	0.8836	0.8147	0.8109
MHDMF	0.9713	0.9234	0.9550
GDCL-NcDA	**0.9844**	**0.9880**	**0.9607**

Different from these traditional similarity network information integration methods (MDA-SKF, DMFMSF and CKA-HGRTMF), GDCL-NcDA does not integrate similarity information through a simple average or linear weighting strategy. It automatically learns the information of each similarity network through depth graph learning and effectively distinguishes the contribution of different similarity information to the prediction task through the attention mechanism. The GDCL-NcDA framework can integrate multi-source similarities in a more reasonable way of calculating. Different from the multi-stage methods (DMFMSF and CKA-HGRTMF), our framework takes an end-to-end approach for data training and prediction. It enables the model to automatically learn relevant and discriminative features from the raw input data. Instead of relying on handcrafted features, the model can effectively extract representations and patterns directly from the data, potentially capturing more intricate and nuanced information. Furthermore, it optimizes all the model parameters jointly, considering the entire pipeline from input to output. This holistic optimization can lead to improved performance as the model can adapt its internal representations and decision-making processes based on the end objective, rather than optimizing individual components separately. Different from the graph learning-based methods (NIMCGCN and MMGCN), this framework utilizes more information from larger MHNs and captures richer and more comprehensive representations. Furthermore, it uses the attention mechanism to strengthen the feature of nodes within the similarity network and the contribution between different similarity networks. GDCL-NcDA can effectively integrate information from multiple sources and improve the overall understanding of the data. We use contrastive learning in this framework to extract semantically meaningful representations by maximizing the similarity between positive pairs and minimizing the similarity between negative pairs. This encourages the framework to focus on capturing essential features and discarding irrelevant or noisy information, resulting in rich and informative representations that can generalize well to downstream tasks. Different from the DMF-based methods (DMFCDA and DMFMSF), our GDCL-NcDA decreases the false-negative of the original associations, which MF relies on. We further integrate more information as additional data into the reconstructed graph. Multi-source information often provide complementary information about the data, capturing different aspects or modalities. Contrastive learning can be used to reduce the need for large amounts of labeled data in the target domain, also reducing the impact of false-negative accordingly. These can improve the ability of GDCL-NcDA to generalize and handle complex patterns and variations. In brief, GDCL-NcDA is feasibility and the effectiveness in underlying ncRNA-disease associations identification, which can be verified by the comparison results thereinbefore.

### Ablation experiments

#### Performance of GDCL-NcDA and its variants

In this section, we illustrate whether the integration of deep graph learning, DMF and contrastive learning within the GDCL-NcDA framework is necessary for the ncRNA-disease associations identification task. We carry out an ablation experiment by split and recombination of our framework. The experiment is conducted under 5CV.

The variant methods we framed include GDCL-NcDA, GDCL-NcDA$\_$GCNII, GDCL-NcDA$\_$GATGCNII, GDCL-NcDA$\_$DMF, GDCL-NcDA$\_$GCNII+DMF, and GDCL-NcDA$\_$GCNII+DMF+CL.

- GDCL-NcDA$\_$GCNII denotes that GCNII and channel attention are only performed to extract and strengthen the embeddings for final identification task.
- GDCL-NcDA$\_$GATGCNII denotes that GAT and GCNII are only performed to enhance and generate the embeddings for final identification task.
- GDCL-NcDA$\_$DMF denotes that DMF is only used for final identification task without any additional information.
- GDCL-NcDA$\_$GCNII+DMF denotes that GCNII used first to reconstruct the association graph, and then, DMF used for final identification task based on the reconstructed graph.
- GDCL-NcDA$\_$GCNII+DMF+CL denotes that GCNII used first to reconstruct the association graph. Then, DMF used to generate predicted graph. The CL used to obtain the loss between the reconstructed graph and predicted graph, which used to update and optimize the entire framework.

As demonstrated in the Table 5, the results of GDCL-NcDA and its variant methods. GDCL-NcDA can attain supreme performance among all methods. For GDCL-NcDA$\_$GCNII and GDCL-NcDA$\_$GATGCNII methods, the latter uses attention mechanism in each similarity network. This result demonstrates that enhancing the features within each similarity network is useful to the identification task. For GDCL-NcDA$\_$GCNII, GDCL-NcDA$\_$DMF and GDCL-NcDA$\_$GCNII+DMF methods, the last one combines the GDCL-NcDA$\_$GCNII and the GDCL-NcDA$\_$DMF. This result demonstrates that associations reconstruction can reduce some real false-negative in original associations. For GDCL-NcDA$\_$GCNII+DMF and GDCL-NcDA$\_$GCNII+DMF+CL methods, the latter adds contrastive loss in framework. This result demonstrates that contrastive learning between GCNII and DMF can be conducive to improve generalization and performance of framework. GDCL-NcDA accomplishes the brilliant performance among these variants, which illustrates the essentials of each component within GDCL-NcDA.

**Table 5:** Performance of GDCL-NcDA and its variants on miRNA-disease MHN

Methods	AUC	AUPRC	F1-score	Recall	Precision
GDCL-NcDA$\_$GCNII	0.8761	0.8810	0.8096	0.8508	0.7736
GDCL-NcDA$\_$GATGCNII	0.8838	0.8940	0.8173	0.8477	0.7906
GDCL-NcDA$\_$DMF	0.8556	0.8661	0.8096	0.8508	0.7736
GDCL-NcDA$\_$GCNII+DMF	0.9720	0.9628	0.9247	0.9153	0.9347
GDCL-NcDA$\_$GCNII+DMF+CL	0.9741	0.9783	0.9328	0.9382	0.9278
GDCL-NcDA	**0.9761**	**0.9806**	**0.9394**	**0.9352**	**0.9439**

#### Performance of GDCL-NcDA on different heterogeneous networks

To show the benefit of using information of larger MHNs, we perform another ablation experiment by leveraging different MHNs used in the GDCL-NcDA framework. All the numerical experiments are carried out under the same number of iteratives and 5CV. In the Table 6, there are all results from the associations between miRNAs, circRNAs, lncRNAs, and their corresponding diseases and genes. These results demonstrate whether the integration of diverse interaction information is beneficial for ncRNA-disease associations identification. GDCL-NcDA achieves outstanding performance by performing on larger MHNs. GDCL-NcDA is more powerful by adding multiple interaction information.

**Table 6:** Performance of GDCL-NcDA on different MHNs

Networks	AUC	AUPRC
miRNA-disease	0.9357	0.9561
miRNA-gene-disease	**0.9761**	**0.9806**
circRNA-disease	0.9455	0.9508
circRNA-gene-disease	**0.9849**	**0.9890**
lncRNA-disease	0.8983	0.9079
lncRNA-gene-disease	**0.9382**	**0.9515**

### Parameter analysis of GDCL-NcDA

In this section, we conduct an experiment analyzing some parameters within the GDCL-NcDA framework to demonstrate their impact. This experiment is under 5CV. In the following, only one parameter is varied to test its effect while the others are fixed.

#### GCNII layer

**Fig. 3:**
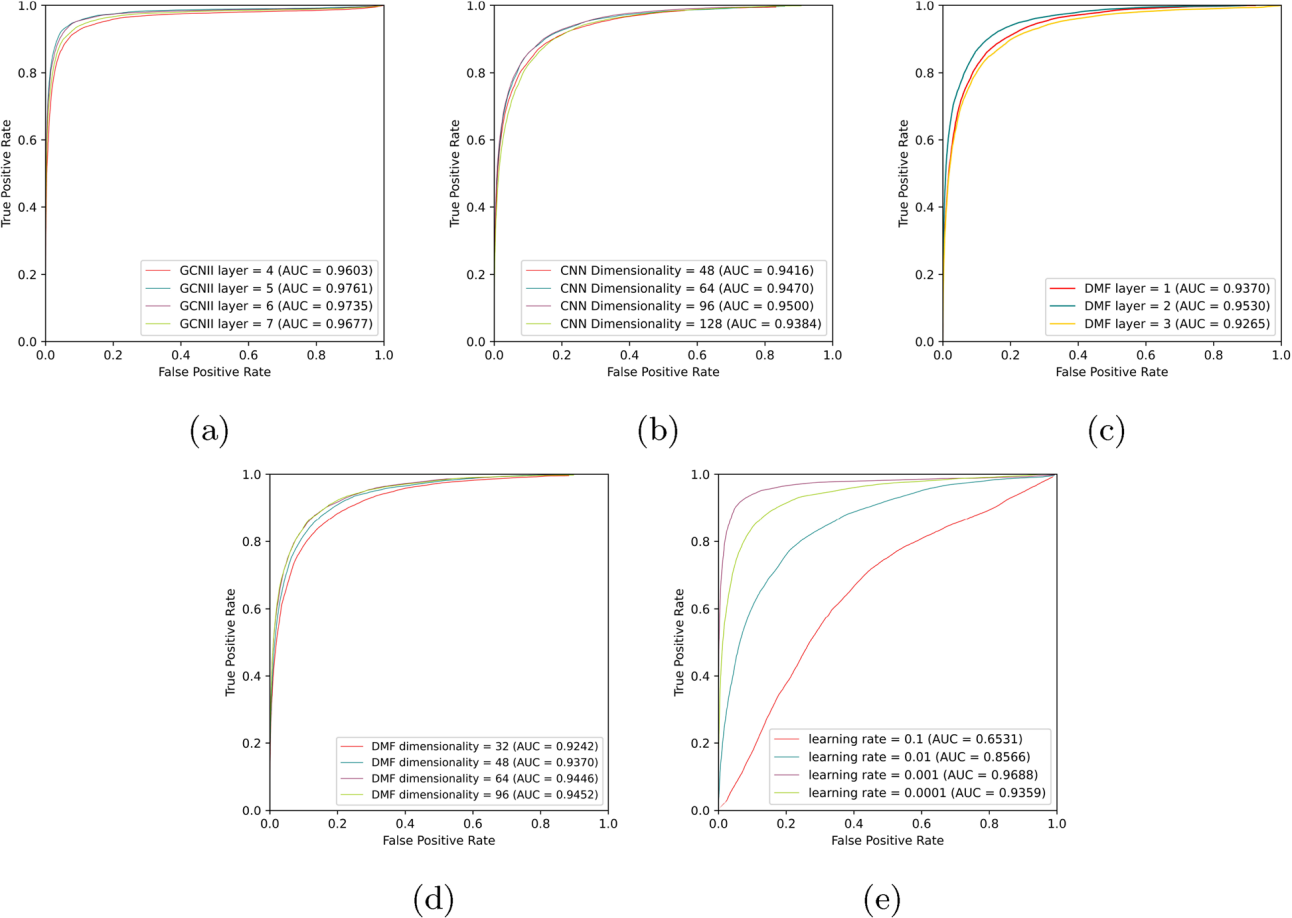
The AUC for parameter analysis of GDCL-NcDA on miRNA-disease MHN

We utilize GCNII to obtain multi-source embeddings for ncRNA and disease. The number of GCNII layer *l* is selected in $\{4, 5, 6, 7\}$. As shown in Fig. 3(a), there is a small influence on GDCL-NcDA performance when the GCNII layer number changes. When the layer number is 5, we obtain optimal performance. In the network topology of biological entities, the biological significance will be greatly reduced if the distance between two biological entities is too far.

#### Dimensionality of CNN features

The CNN dimensionality determines the size of final embeddings of ncRNA and disease. After generating these embeddings, the framework will implement the succeeding association graph reconstruction task. The CNN dimensionality is selected from $\{48, 64, 96, 128\}$, shown in Fig. 3(b), it can be discovered that the performance of GDCL-NcDA has tiny changes under different dimensionalities. When CNN dimensionality is 96, we obtain optimal performance.

#### DMF layer

The number of DMF layers will directly affect the result of the identification task. The number of DMF layers is selected from $\{1, 2, 3\}$, when it is 2, we obtain optimal performance, as shown in Fig. 3(c).

#### Dimensionality of DMF feature

We use DMF to extract the features of ncRNA-disease associations via potential features in a common low-dimensional space. Therefore, the DNF dimensionality of potential features is crucial for the predicted graph generated. The DMF dimensionality for potential feature is selected from $\{32, 48, 64, 96\}$, when DMF dimensionality is 96, we obtain optimal performance, as shown in Fig. 3(d).

#### Learning rate

As learning rate can control the size of step for gradient descent, it be a significant hyper-parameter for deep learning. Step size is one of the factors that determine whether the algorithm can reach the optimal solution. A bad learning rate can lead to a number of problems. For example, the model is unstable and unable to converge, easily falls into local optimal, slow convergence and other problems. The learning rate is selected in $\{0.1, 0.01, 0.001, 0.0001\}$, when it is 0.001, we obtain optimal performance, as shown in Fig. 3(e).

### Case studies

We illustrate the ability of GDCL-NcDA with case studies for ncRNA-disease associations identification. The performance of case studies for GDCL-NcDA is further assessed by two specific diseases for miRNA, circRNA, and lncRNA. More explicitly, we choose diverse cancers, such as lung neoplasms and brain cancer for miRNA, cervical cancer and breast cancer cancer for circRNA, and ovarian cancer and kidney cancer for lncRNA. In this work, we rank the predicted score of unknown associations from those MHNs.

Table 7 displays the top-10 candidate miRNAs, and further proved the predicted associations by performing ① dbDEMC [64], ② HDMM v3.2 [65], and ③ MNDR2.0 [66]. The HDMM v3.2 database is the updated version of the HMDD v2.0 database [31], from which we download the positive set for our MHN of miRNA-disease. More specifically, the top-10 candidate miRNAs we identified, which did not appear in HDMM v2.0 but found validation in HDMM v3.2, further illustrate the effectiveness of our GDCL-NcDA framework.

**Table 7:** The top 10 candidate miRNAs identified by GDCL-NcDA for (1) lung neoplasms and (2) brain cancer

Ranking	miRNAs of (1)	Evidence
1	hsa-mir-375	①/②
2	hsa-mir-376a-1	①/②
3	hsa-mir-376a-2	①/②
4	hsa-mir-376b	①
5	hsa-mir-376c	①
6	hsa-mir-377	①/②/③
7	hsa-mir-379	①
8	hsa-mir-381	①/②
9	hsa-mir-383	①/②
10	hsa-mir-24-2	①/②
Ranking	miRNAs of (2)	Evidence
1	hsa-mir-192	①/②
2	hsa-mir-205	①/②
3	hsa-mir-181c	①/②
4	hsa-mir-143	①/②
5	hsa-let-7a-2	①/②
6	hsa-let-7a-3	①/②
7	hsa-let-7a-1	②
8	hsa-mir-494	①/②
9	hsa-mir-183	①/②
10	hsa-mir-92b	①/②

Table 8 displays the top-10 candidate circRNAs, and further verified the predicted associations by utilizing ④ circMine [67], and ⑤ Lnc2Cancer3.0 [68]. Table 9 displays the top-10 candidate lncRNAs, and further proved the predicted associations by employing ⑥ LncRNADisease v2.0 [69], ③MNDR2.0, and ⑤ Lnc2Cancer3.0.

**Table 8:** The top 10 candidate circRNAs identified by GDCL-NcDA for (1) cervical cancer and (2) breast cancer

Ranking	circRNAs of (1)	Evidence
1	hsa$\_$circ$\_$0002113	④
2	hsa$\_$circ$\_$0061893	Unconfirmed
3	hsa$\_$circ$\_$0004771	③/④
4	hsa$\_$circ$\_$0043138	④
5	hsa$\_$circ$\_$101396	Unconfirmed
6	hsa$\_$circ$\_$103783	Unconfirmed
7	hsa$\_$circ$\_$102533	Unconfirmed
8	hsa$\_$circ$\_$102470	Unconfirmed
9	hsa$\_$circ$\_$0023984	④
10	hsa$\_$circ$\_$0007534	③/⑤
Ranking	circRNAs of (2)	Evidence
1	hsa$\_$circ$\_$0000064	Unconfirmed
2	hsa$\_$circ$\_$0014717	④
3	hsa$\_$circ$\_$102231	Unconfirmed
4	hsa$\_$circ$\_$103595	Unconfirmed
5	hsa$\_$circ$\_$104964	Unconfirmed
6	hsa$\_$circ$\_$0000615	③/④/⑤
7	hsa$\_$circ$\_$0030045	④
8	hsa$\_$circ$\_$001937	Unconfirmed
9	hsa$\_$circ$\_$0001417	④
10	hsa$\_$circ$\_$100290	④

**Table 9:** The top 10 candidate lncRNAs identified by GDCL-NcDA for (1) ovarian cancer and (2) kidney cancer

Ranking	lncRNAs of (1)	Evidence
1	BDNF-AS	⑥
2	BGLT3	Unconfirmed
3	BOK-AS1	⑥
4	CAHM	⑥
5	CASC15	③/⑤/⑥
6	CASC2	③/⑥
7	CASC22	⑥
8	CASC9	③
9	CBR3-AS1	⑥
10	CCAT1	③/⑤/⑥
Ranking	lncRNAs of (2)	Evidence
1	PTPRD-AS1	Unconfirmed
2	DGCR5	⑥
3	SNHG11	⑥
4	NCRUPAR	Unconfirmed
5	MESTIT1	⑥
6	HIF1A-AS1	③/⑥
7	ESRG	⑥
8	HOTAIRM1	⑤
9	CCAT1	⑤
10	PCAT18	Unconfirmed

## Conclusion

The central dogma of molecular biology describes how genetic information is transmitted through RNA to the corresponding protein. As ncRNAs do not involved in transcription of proteins, they are treated as the transcriptional noise. With the development of biotechnology, ncRNA has attracted wide attention. For the past few years, increasing experimental skills demonstrate that ncRNA is badly related to the development of diverse human diseases. However, the relationship verified by wet experimental skills is not sufficient to further explore the pathogenic mechanism at the molecular level of disease. Therefore, it is essential to develop the computational method for studying the ncRNA-disease associations.

In this work, we develop a novel end-to-end framework called GDCL-NcDA, which accomplishes brilliant performance on three MHNs, including three varieties of ncRNA (miRNA, circRNA, and lncRNA). Different from previous works, we construct multiple MHNs of three varieties ncRNA, disease, and gene, and use deep graph learning and multiple attention mechanisms to reconstruct associations between ncRNAs and diseases, on which DMF to generate the predicted associations based. Furthermore, we add contrastive learning between reconstructed associations and predicted associations to improve the generalization of our framework. In practice, the feasibility and availability of GDCL-NcDA is also proved by our following experiments.

GDCL-NcDA can not only efficiently make use of restricted verified associations to predict latent relation, but also fuse multi-source information of MHNs to weaken the false-negative of ncRNA-disease associations accountably. The experimental results account for that GDCL-NcDA obtains outstanding performance among state-of-the-art methods we compared under 5CV and 10CV. Additionally, diverse ablation experiments show evidence of the availability of different modules within GDCL-NcDA and the efficacy for MHNs construction. Finally, we construct case studies to further give evidence of the potential ability of GDCL-NcDA in identifying the underlying candidate disease-related ncRNAs.

## Data Availability

For miRNA-disease, the positive set of miRNA-disease associations is downloaded from the HMDD v2.0 database [31]: http://cmbi.bjmu.edu.cn/hmdd. The miRNA-gene associations are downloaded from the miRWalk2.0 database [32]: http://mirwalk.umm.uni-heidelberg.de/. The disease-gene associations are downloaded from DisGeNET [33]: https://www.disgenet.org/. For circRNA-disease, we download the positive associations of circRNA-disease from CircR2Disease database [35]: http://bioinfo.snnu.edu.cn/CircR2Disease/, the circRNA-gene associations from http://cssb2.biology.gatech.edu/knowgene/search.html, and the disease-gene associations from http://cssb2.biology.gatech.edu/knowgene/. For lncRNA-disease, we obtain the lncRNA-disease positive linkages from LncRNADisease database [36]: https://www.cuilab.cn/lncrnadisease, the lncRNA-gene linkages from lncReg database [37]: https://www.lncrnablog.com/tag/lncreg/, and the disease-gene linkage from DisGeNet database. All Disease semantic similarity are downloaded from MeSH [34]: http://www.nlm.nih.gov. The code of GDCL-NcDA is provided on GitHub (https://github.com/AINING96/GCL_NcDA).
